# The Antileishmanial Activity of Eugenol Associated with Lipid Storage Reduction Rather Than Membrane Properties Alterations

**DOI:** 10.3390/molecules28093871

**Published:** 2023-05-04

**Authors:** Kristelle Hughes, Thanh Binh Le, Patrick Van Der Smissen, Donatienne Tyteca, Marie-Paule Mingeot-Leclercq, Joëlle Quetin-Leclercq

**Affiliations:** 1 Pharmacognosy Research Group, Louvain Drug Research Institute, Université Catholique de Louvain, Avenue E. Mounier 72, B1.72.03, B-1200 Brussels, Belgium; kristelle.hughes@uclouvain.be (K.H.); binhnt@hup.edu.vn (T.B.L.); 2 CELL Unit and PICT Imaging Platform, de Duve Institute, Université Catholique de Louvain, Avenue Hippocrate 75, B1.75.05, B-1200 Brussels, Belgium; patrick.vandersmissen@uclouvain.be (P.V.D.S.); donatienne.tyteca@uclouvain.be (D.T.); 3 Cellular and Molecular Pharmacology Unit (FACM), Louvain Drug Research Institute, Université Catholique de Louvain, Avenue E. Mounier 73, B1.73.05, B-1200 Brussels, Belgium; marie-paule.mingeot@uclouvain.be

**Keywords:** eugenol, *Leishmania*, mode of action, lipid droplets, acidocalcisomes, membrane permeability, membrane fluidity

## Abstract

Leishmaniasis is a neglected tropical disease that still infects thousands of people per year throughout the world. The occurrence of resistance against major treatments for this disease causes a healthcare burden in low-income countries. Eugenol is a phenylpropanoid that has shown in vitro antileishmanial activity against *Leishmania mexicana mexicana* (*Lmm*) promastigotes with an IC_50_ of 2.72 µg/mL and a high selectivity index. Its specific mechanism of action has yet to be studied. We prepared large unilamellar vesicles (LUVs), mimicking *Lmm* membranes, and observed that eugenol induced an increase in membrane permeability and a decrease in membrane fluidity at concentrations much higher than IC_50_. The effect of eugenol was similar to the current therapeutic antibiotic, amphotericin B, although the latter was effective at lower concentrations than eugenol. However, unlike amphotericin B, eugenol also affected the permeability of LUVs without sterol. Its effect on the membrane fluidity of *Lmm* showed that at high concentrations (≥22.5× IC_50_), eugenol increased membrane fluidity by 20–30%, while no effect was observed at lower concentrations. Furthermore, at concentrations below 10× IC_50_, a decrease in metabolic activity associated with the maintenance of membrane integrity revealed a leishmaniostatic effect after 24 h of incubation with *Lmm* promastigotes. While acidocalcisomes distribution and abundance revealed by *Trypanosoma brucei* vacuolar H^+^ pyrophosphatase (TbVP1) immunolabeling was not modified by eugenol, a dose-dependent decrease of lipid droplets assessed by the Nile Red assay was observed. We hereby demonstrate that the antileishmanial activity of eugenol might not directly involve plasma membrane sterols such as ergosterol, but rather target the lipid storage of *Lmm*.

## 1. Introduction

Leishmaniasis is a series of vector-borne diseases, transmitted through the bite of a sandfly infected by *Leishmania* parasites. This disease is endemic to several regions in the world, including some developed countries [1]. In 2020, there were over 208,000 new cases of cutaneous leishmaniasis reported worldwide and nearly 13,000 new visceral leishmaniasis cases [2]. Current therapies include sometimes long, painful, and toxic treatments such as pentavalent antimonial, liposomal amphotericin B, miltefosine, or paromomycin [3]. Furthermore, each therapy is effective for a limited number of *Leishmania* species and, thus, specific forms of the disease.

Liposomal amphotericin B is used to treat visceral leishmaniasis [3]. Its leishmanicidal activity is mediated through the formation of pores in the *L. mexicana* and *L. donovani* promastigote plasma membrane as it binds membrane sterols, namely ergosterol, and cholesterol to a lesser extent [4,5]. Formulations such as Kalsome TM10 were shown to depolarize the mitochondrial membrane, cause ATP levels depletion, and increase reactive oxygen species (ROS) production [6].

Miltefosine is the only oral treatment used against cutaneous leishmaniasis as well as visceral leishmaniasis in endemic regions such as South America and India [7]. The drug is an alkylphosphocholine that has been shown to have several targets in *Leishmania* cells, although its mechanism of action is not fully elucidated [8,9,10,11]. 

In addition to therapy efficacy in specific forms of the disease, only, recent emergence of resistant strains and/or relapses after treatment have been reported for both treatments. For instance, mutations to the enzyme sterol 14α-demethylase, leading to a decrease in plasma-membrane ergosterol levels [12], and higher levels of multidrug resistance 1 (MDR1), causing greater amphotericin B efflux [13], have been shown to decrease the efficacy of amphotericin B treatments. Many in-laboratory miltefosine-resistant strains obtained through several mutations, lead to drug efflux or a reduction in uptake, and an increase in oxidative stress [14]. However resistant strains have not yet been discovered in clinical isolates against miltefosine, though several relapses after treatment have nonetheless been documented [15,16]. 

In this context, eugenol could represent a potentially useful molecule. An eugenol oil-in-water emulsion showed beneficial immunomodulatory activity in mice infected with *L. donovani* [17]. Indeed, after 10 days of treatment with the eugenol emulsion, the hepatic and splenic parasites load was significantly decreased compared to untreated infected mice, and the host defense was stimulated as evidenced by an increase in nitric oxide, IFN-γ, and IL-2 levels, a decrease in IL-4 and IL-10 levels, as well as an increase in CD4+ and CD8+ T cells. Nonetheless, the mechanism of action of eugenol on *Leishmania* promastigotes has not yet been elucidated. Previous research conducted in our group showed that eugenol has an antileishmanial activity of 2.72 µg/mL (16.59 µM) against *Leishmania mexicana mexicana* (*Lmm*) promastigotes, with a selectivity index above 10 against the WI38 human fibroblasts and the J774 macrophage mouse-derived cell line [18]. 

The aim of the present study was to better characterize the mechanism of action of eugenol on *Leishmania,* other than its immunomodulatory activity. Amphotericin B and miltefosine were used as reference drugs in the different assays, not only to assess the magnitude of the activity but also to better determine underlying mechanisms of action. To this aim, morphological changes to *Lmm* promastigotes after treatment with increasing concentrations of eugenol during 24 h and 72 h were followed by scanning electron and fluorescence microscopy. Membrane permeability was evaluated on liposomes (LUVs mimicking *Lmm* membranes) by following calcein release. Membrane fluidity was studied on both LUVs and *Leishmania* promastigotes by measuring changes in 1,6-diphenyl-1,3,5-hexatriene (DPH) anisotropy. Apoptosis in *Lmm* populations was followed by FACS analysis of the calcein-acetoxymethyl ester (AM)/propidium iodide (PI) dye staining. Metabolic activity through NADH/NAD+ and cytochrome c oxidase was assessed in *Leishmania* by using the Alamar blue assay. Finally, in an effort to elucidate a potential target of eugenol in the metabolism of *Lmm*, the abundance of acidocalcisomes and lipid droplets, respectively, involved in pH and Ca^2+^ homeostasis and energy storage, were quantified in *Lmm* promastigotes using fluorescence microscopy for anti-*Trypanosoma brucei* vacuolar H^+^ pyrophosphatase (TbVP1) and Nile Red labeling.

Altogether, the results show that eugenol possesses a mechanism of action that differs from that of the two known drugs amphotericin B and miltefosine, by targeting lipid storage at concentrations below 10× IC_50_, leading to a stunt in cell growth while maintaining membrane integrity. At concentrations above 25× IC_50_, eugenol causes membrane disruption though without selectively targeting membrane sterols. These data give insight into the mode of action of eugenol to mediate its antiparasitic activity.

## 2. Results

### 2.1. Eugenol Induces Round Shape of Lmm Promastigotes

**Figure 1:**
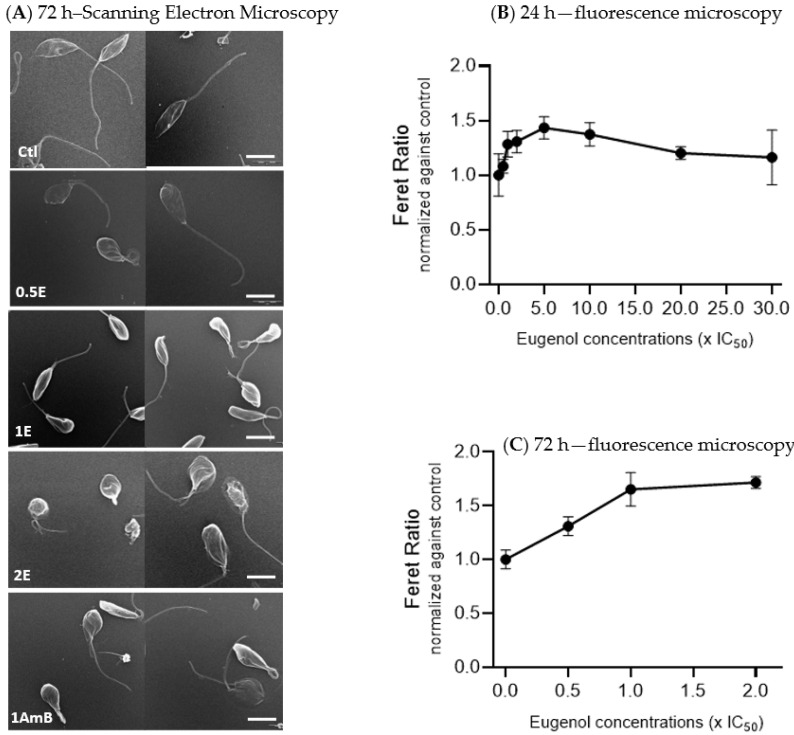
Eugenol induces ultrastructural changes in *Leishmania mexicana mexicana* promastigotes, as revealed by scanning electron and confocal microscopy. (**A**) Representative images in scanning electron microscopy of *Lmm* treated for 72 h with 0.01% DMSO as control (Ctl), eugenol at 0.5–2× IC_50_ (0.5–2E), and amphotericin B at IC_50_ (1AmB). Scale bars, 5 µm. (**B**,**C**) Quantification of *Leishmania* morphology by Nile Red labeling in fluorescence microscopy. *Lmm* promastigotes were treated during 24 h (**B**) and 72 h with eugenol (**C**). The Feret ratio was calculated from the minimum Feret diameter divided by the maximum Feret diameter. Values ± SD of 2 independent experiments.

To evaluate the general effect of eugenol on *Lmm*, promastigotes were treated for 72 h with 0.01% DMSO (Ctl) or three concentrations of eugenol (0.5× IC_50_, IC_50_, and 2× IC_50_) or amphotericin B (at IC_50_). Images were recorded by using scanning electron microscopy (SEM) (Figure 1A). 

SEM analysis of untreated *Leishmania* promastigotes showed elongated bodies with long flagella. Promastigotes treated with eugenol at 0.5× IC_50_ appeared more swollen and at IC_50_ and 2× IC_50_ showed alterations in both shape and size, as observed with gradually rounding promastigote bodies and loss or shortening of their flagella. At 2× IC_50_, folds appeared on the body of the *Lmm* and their flagella became stumpy. Amphotericin B, at IC_50_, also showed more round-shaped bodies compared to the control. 

Feret ratio values, which describe the shape of the promastigotes (the higher the value, the rounder the object), were calculated. Data revealed an increase of 1.4 fold, reached between 5 and 10× IC_50_ of eugenol, followed by a decrease back to values comparable to the control at 20 and 30× IC_50_ of eugenol after 24 h of treatment (Figure 1B). After 72 h of treatment, a dose-dependent increase of the Feret ratio was observed till 1.7 fold at 2× IC_50_ (Figure 1C). 

Altogether, these results indicate that eugenol induced rounding of *Lmm* promastigotes bodies, similar to amphotericin B.

### 2.2. Eugenol Induces Milder Membrane Permeability in LUVs Than Amphotericin B

**Figure 2:**
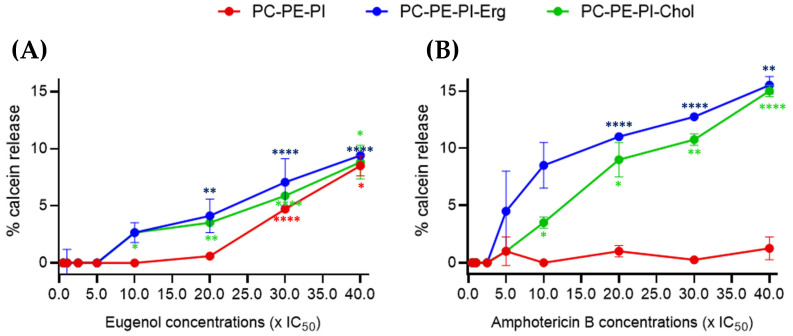
The release of calcein induced by eugenol is lower than that of amphotericin B with differences in sterol dependency. Three LUVs, without sterol (PC:PE:PI; red), with ergosterol (PC:PE:PI:Erg; blue), and with cholesterol (PC:PE:PI:Chol; green), were incubated with increasing concentrations (0.5–40× IC_50_) of eugenol (**A**) or amphotericin B (**B**) during 15 min. Fluorescence was measured to follow calcein release and data were expressed in % of calcein release with 0.1% Triton X100, which induces 100% leakage. The values ± SD were obtained in triplicates for three independent experiments. One-way ANOVA with Dunnett’s post tests were performed to compare control versus compound-treated LUVs, * *p* < 0.05, ** *p* < 0.01, **** *p* < 0.0001.

To then evaluate the potential effect of eugenol on membranes, we examined changes in the permeability of large unilamellar vesicles (LUVs) mimicking *Lmm* membranes. Three LUVs compositions were tested. All were composed of phosphatidylcholine (PC), phosphatidylethanolamine (PE), and phosphatidylinositol (PI) but differential sterol content: PC:PE:PI, PC:PE:PI:Cholesterol, or PC:PE:PI:Ergosterol. Each model was treated with increasing concentrations of eugenol and amphotericin B and the calcein leakage was followed (Figure 2). 

Between 0.5 and 5× IC_50_, eugenol showed no effect on LUVs, regardless of the liposome composition. At 10× IC_50_, a low calcein leakage of 2.7% was observed in ergosterol- and cholesterol-containing LUVs. At 30× IC_50_, a calcein release of 4.7% was observed in sterol-free LUVs. At 40× IC_50_, ~9% of calcein was released for all three liposome membrane compositions (Figure 2A). 

Amphotericin B was used as a reference drug since its interactions with ergosterol in *Lmm* plasma membranes are well known [4,19,20]. No effect was observed until 2.5× IC_50_. A ~4% calcein leakage was observed starting from 5× IC_50_ for ergosterol-containing LUVs and from 10× IC_50_ for cholesterol-containing LUVs, while, as expected, no leakage was observed for the LUVs without sterol even at the highest-tested concentration. A gradual increase was observed for both sterol-containing compositions until 40× IC_50_ (Figure 2B). 

All in all, these data show that eugenol increased LUV permeability at two-times-higher IC_50_ concentrations than amphotericin B. Furthermore, there was seemingly a slight difference in effect between sterol- and nonsterol-containing LUVs treated with eugenol, though no difference between ergosterol- and cholesterol-containing LUVs, suggesting a different mechanism of action than amphotericin B.

### 2.3. Eugenol Similarly Decreases Membrane Fluidity in Cholesterol and Ergosterol LUVs

Then, using the same eugenol and amphotericin B concentrations as in the permeability assay, we evaluated the membrane fluidity through the measurement of DPH anisotropy. An increase in DPH anisotropy indicates a decrease in membrane fluidity [21]. Only the models containing sterols were kept as there was no significant effect on permeability after treatment in the sterol-free model for amphotericin B. 

**Figure 3:**
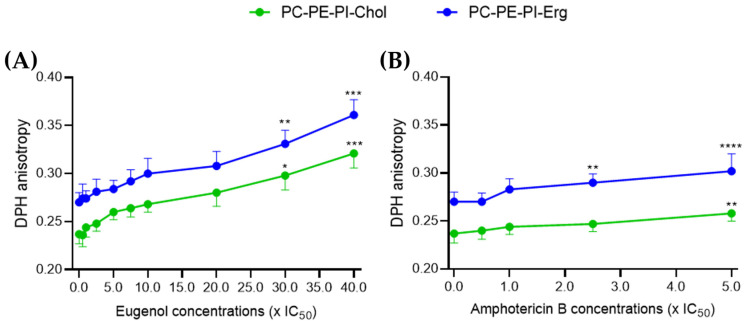
Eugenol similarly decreases membrane fluidity of ergosterol- and cholesterol-containing LUVs. PC:PE:PI:Erg (blue) and PC:PE:PI:Chol (green) LUVs were mixed with DPH at a molar ratio of 1:200 (DPH:lipid) and were incubated for 30 min in the dark with 0.01% DMSO as control, and increasing concentrations of eugenol between 0.5–40× IC_50_ (**A**) and of amphotericin B between 0.5–5× IC_50_ (**B**). Fluorescence was measured to determine DPH anisotropy. The curves represent values ± SD obtained for triplicates from three independent experiments. One-way ANOVA with Dunnett’s post tests were performed to compare the control versus the compound-treated LUVs, * *p* < 0.05, ** *p* < 0.01, *** *p* < 0.001, and **** *p* < 0.0001.

Between 0.5 and 20× IC_50_, eugenol showed no significant effect on DPH anisotropy compared to the control. However, starting from 30× IC_50_, eugenol induced a 30 to 35% dose-dependent decrease in membrane fluidity in both ergosterol- and cholesterol-containing LUVs (Figure 3A). 

A significant 7% decrease in membrane fluidity was observed after treatment with amphotericin B at 2.5× IC_50_ and 12% at 5× IC_50_ in ergosterol-containing LUVs. This is in agreement with previous results that a colloidal suspension containing 45% amphotericin B was shown to decrease the plasma membrane fluidity of *L. amazonensis* promastigotes by increasing EPR spin labeling in a dose-dependent manner [22]. For cholesterol-containing LUVs, only a 9% decrease in fluidity was observed at 5× IC_50_ (Figure 3B).

Altogether, these results on LUVs indicate that, like amphotericin B, eugenol was able to decrease the membrane fluidity, whether in the presence of ergosterol or cholesterol. However, concentrations of eugenol 15 times higher than those used for amphotericin B are required to show a significant decrease in membrane fluidity. 

### 2.4. Eugenol Increases the Fluidity of Lmm Membranes at Higher Concentrations Than Amphotericin B

To enhance the biological relevance of our results and ensure that the differences only in concentrations but not in activity between eugenol and amphotericin B did not result from the simple composition of LUVs, we directly measured DPH anisotropy on the *Lmm* membranes [23,24].

**Figure 4:**
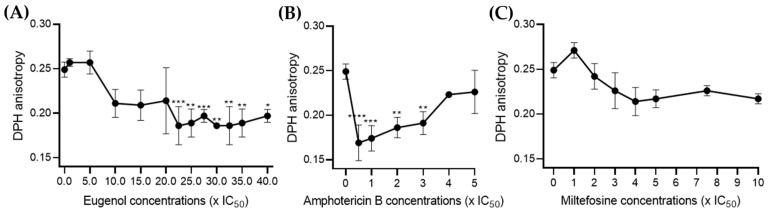
Eugenol increases the fluidity of *Lmm* membranes at concentrations strongly higher than amphotericin B while miltefosine shows no effect. *Lmm* promastigotes at a density of 10^7^ cells/mL were treated for 24 h with 0.1% DMSO as control, eugenol at 0.5–40× IC_50_ (**A**), or amphotericin B at 0.5–5× IC_50_ (**B**), or miltefosine at 1–10× IC_50_ (**C**). Fluorescence was measured to determine DPH anisotropy. The curves represent values ± SD obtained for triplicates from three independent experiments. Significance levels were determined by one-way ANOVA followed Dunnett’s post tests to compare control versus compound-treated plasma membranes * *p* < 0.05, ** *p* < 0.01, *** *p* < 0.001, and **** *p* < 0.0001.

For eugenol, no significant effect was observed below 20× IC_50_. Between 22.5 and 40× IC_50_, eugenol increased membrane fluidity by 20–30%, which is contrary to its activity in LUVs (Figure 4A). Amphotericin B also displayed an increase in membrane fluidity below 3× IC_50_, while the DPH value increased dose dependently from 0.5 to 5× IC_50_ (Figure 4B). In contrast, miltefosine showed no significant effect on membrane DPH at any of the concentrations tested (Figure 4C). 

In sum, the above data indicate that eugenol induced similar changes to membrane permeability and fluidity than amphotericin B, though alterations were seen at higher concentrations and were less sterol-dependent, which could suggest a different mechanism of action.

### 2.5. Eugenol Exerts Its Leishmanicidal Effect with Kinetic and Cell-Death Mechanisms Different from Those of Amphotericin B and Miltefosine

To further assess the mode of action of eugenol, we studied the cell-death mechanisms induced by this compound in comparison with amphotericin B and miltefosine. 

Calcein-acetoxymethyl ester (AM)/propidium iodide (PI) double labelling can be used on *Leishmania* to distinguish between living, early apoptotic, late apoptotic, and necrotic cells. Contrary to mammalian cells, calcein-AM has greater difficulty entering into viable *Leishmania* cells with intact membranes, resulting in low-to-no fluorescence (calcein−/PI−) [25,26]. During apoptosis, calcein-AM enters the cells where it is cleaved by esterases into a fluorescent molecule. However, at the early apoptosis stage, membrane modifications with the maintenance of the membrane integrity allow calcein-AM entry but prevent PI entry (calcein+/PI−). During late apoptosis, membrane degradation creates pores in the plasma membrane allowing PI to enter the cells (calcein+/PI+). Finally, in necrotic cells, complete membrane loss leads to PI being solely observed (calcein−/PI+) [25]. 

**Figure 5:**
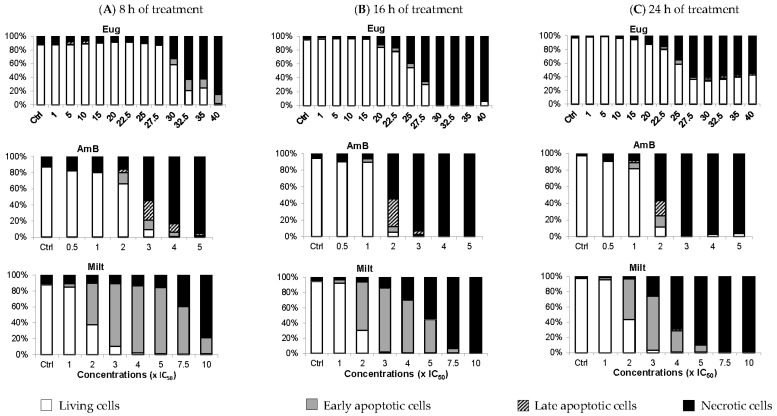
In contrast to miltefosine (Milt) which mainly induces apoptosis, eugenol (Eug) mainly provokes necrosis of Lmm promastigotes as amphotericin B (Amb) but at higher concentrations. Percentage of living (white), early apoptotic (grey), late apoptotic (striped), and necrotic (black) *Lmm* promastigote cells obtained after treatment for 8 h (**A**), 16 h (**B**), and 24 h (**C**) by Eug (1–40× IC_50_) and by AmB (0.5–5× IC_50_), and Milt (1–10× IC_50_). Treated cells were labeled with calcein-AM at 0.1 μM and PI at 3 μM, then incubated for 15 min at room temperature in the dark. Green and red fluorescence emissions were measured for calcein and propidium iodide, respectively, by flow cytometry using a 488 nm excitation. The histogram values are the mean of three independent experiments.

In Figure 5, between 1 and 15× IC_50_, eugenol did not induce apoptosis whether the treatment lasted 8 h, 16 h, or 24 h. Only 3.5% of cells were in the late apoptotic stage at 5 and 10× IC_50_, after 8 h of treatment. At 30 and 32.5× IC_50_, 9.7% and 16.7% of promastigotes were in early apoptosis, respectively, after 8 h of treatment. After 16 h of treatment at these concentrations, over 95% of cells were necrotic, while after 24 h of treatment, the living cell population rose to 35%, with ~5% early and late apoptotic cells combined. The lower percentage of necrotic cells after 24 h of treatment compared to living cells could be due to more cells evolving from a necrotic state to being completely deteriorated, and thus not detected using our double labeling. Alternatively, the *Lmm* doubling time enabled more living cells to multiply, increasing their proportion, or cells started adjusting to the presence of eugenol, which led to more cells in the early apoptotic phase than in the necrotic phase. 

In contrast to eugenol, amphotericin B induced early and late apoptotic cells starting at 1 or 2× IC_50_ according to the duration of treatment. However, it mainly caused necrosis at higher concentrations. Miltefosine was also added as it was already shown to induce apoptosis in *L. tropica* at 10 µM and *L. major* promastigotes at 22 µM after 18 h, 24 h, and 36 h of incubation by flow-cytometry analysis of annexin V [27]. In the case of miltefosine, *Lmm* cells were in their early apoptosis phase starting at 2× IC_50_ for all three treatment durations. Regarding necrosis, the concentration of miltefosine required to induce at least 40% of necrotic cells is inversely proportional to the incubation time.

In conclusion, eugenol induced apoptosis when applied for 8 h at a high concentration (≥30× IC_50_). After 24 h of treatment at these concentrations, eugenol likely induced necrosis, a phenomenon that can be linked to membrane disruption via increased membrane permeability and fluidity. At lower concentrations of eugenol, cells were mainly living and membrane integrity was maintained. We, therefore, focused on evaluating the effect of eugenol on metabolic activity to characterize intracellular events that could be responsible for the antileishmanial activity of the compound.

### 2.6. Eugenol Drastically Reduces Metabolic Activity at Low Concentrations While Maintaining a High Percentage of Living Cells

Alamar Blue is a reagent containing resazurin, a blue dye. Resazurin enters the cell where it is reduced to the highly fluorescent pink-molecule resofurin by enzymes and cofactors such as NADH oxidoreductase or cytochrome *c* oxidase [28,29,30] without impairing mitochondrial respiration. It is used to follow cell viability and metabolic function [31], as it is sensitive to changes in energy or respiratory metabolism [32,33]. Furthermore, NADH/NAD^+^ balance and related enzymes have been shown to be crucial in *Leishmania* survival [34]. The metabolic activity data obtained by the Alamar Blue assay were then plotted in comparison with the percentage of living cells obtained by FACS (see Figure 5).

**Figure 6:**
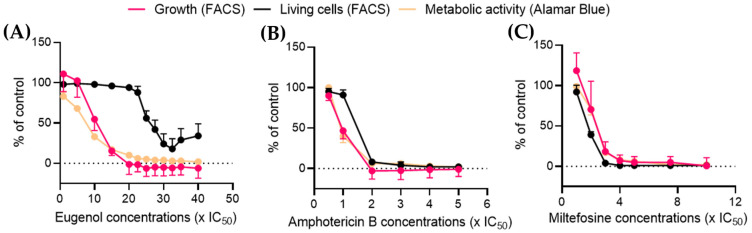
Eugenol reduces the metabolic activity of Leishmania while maintaining a high proportion of living cells in contrast to amphotericin B and miltefosine. Metabolic activity was determined with the Alamar blue assay and cell growth was determined by FACS analysis after 24 h of treatment with eugenol at 0.5–40× IC_50_ (**A**), amphotericin B at 0.5–5× IC_50_ (**B**), and miltefosine at 0.5–10× IC_50_ (**C**). The percentage of living cells was obtained from the FACS experiment described in Figure 5. Values are presented ± SD and were obtained from three independent experiments.

After 24 h of treatment at 10× IC_50_, eugenol reduced the metabolic activity by more than half while simultaneously inducing a 40% loss of *Lmm* division. In contrast, the calcein-AM/PI double labeling showed 95% living parasites at this concentration. The percentage of living cells was reduced by half only at concentrations higher than 25× IC_50_ and reached a plateau at 27.5× IC_50_ (Figure 6A). Amphotericin B and miltefosine both induced a sharp decline in the living-cell population coupled with a dose-dependent reduction of cell growth and metabolic activity (Figure 6B,C). 

These results indicate that contrary to amphotericin B and miltefosine, eugenol induces a leishmanostatic effect at concentrations below 15× IC_50_, which is characterized by a reduction of metabolic activity and cell growth while maintaining living cell populations. 

### 2.7. Eugenol Reduces Lipid Inclusions but Does Not Impair Acidocalcisomes

Since the metabolic activity of *Lmm* measured by the Alamar Blue assay was reduced in the presence of eugenol, we evaluated whether this observation could be linked to an alteration of polyphosphate storage and osmoregulation in acidocalcisomes and/or lipid metabolism via lipid droplets [35,36]. Acidocalcisomes and lipid droplets were therefore assessed after 24 h of treatment with increasing concentrations of eugenol by fluorescence microscopy upon anti-TbVP1 and Nile Red labeling, respectively.

**Figure 7:**
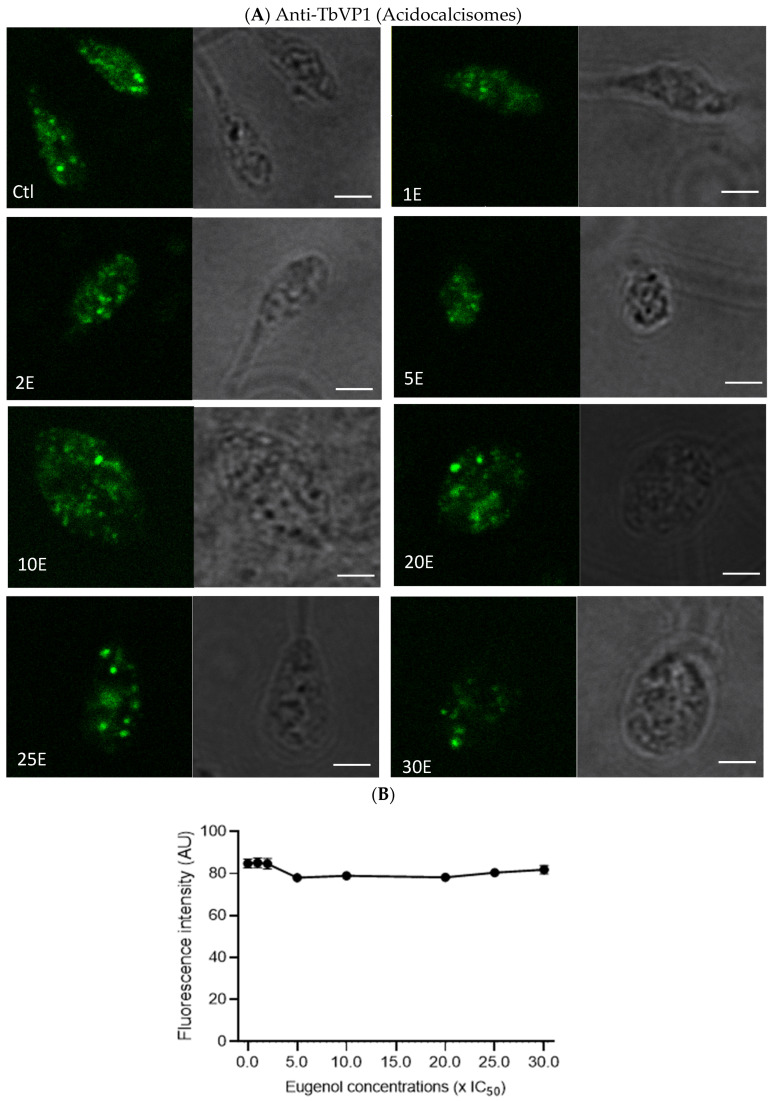
Eugenol does not alter the distribution and abundance of acidocalcisomes, as evidenced by immunolabelling of the vacuolar-type H^+^ pyrophosphatase TbVP1. (**A**) Representative images of acidocalcisomes localization and abundance by anti-TbVP1 immunofluorescence microscopy of *Lmm* promastigotes treated for 24 h with 0.01% DMSO as control (Ctl) and eugenol at 1–30× IC_50_ (1–30E) on the left panel and corresponding brightfield images in right panel. Scale bars, 2 µm. (**B**) Image quantification. Values are presented ± SD and are from one experiment with 4–6 images per condition.

In Figure 7A,B, no obvious effect on acidocalcisomes was noticed after 24 h or 72 h. This suggests that the overall content and number of acidocalcisomes was not impacted by eugenol.

**Figure 8:**
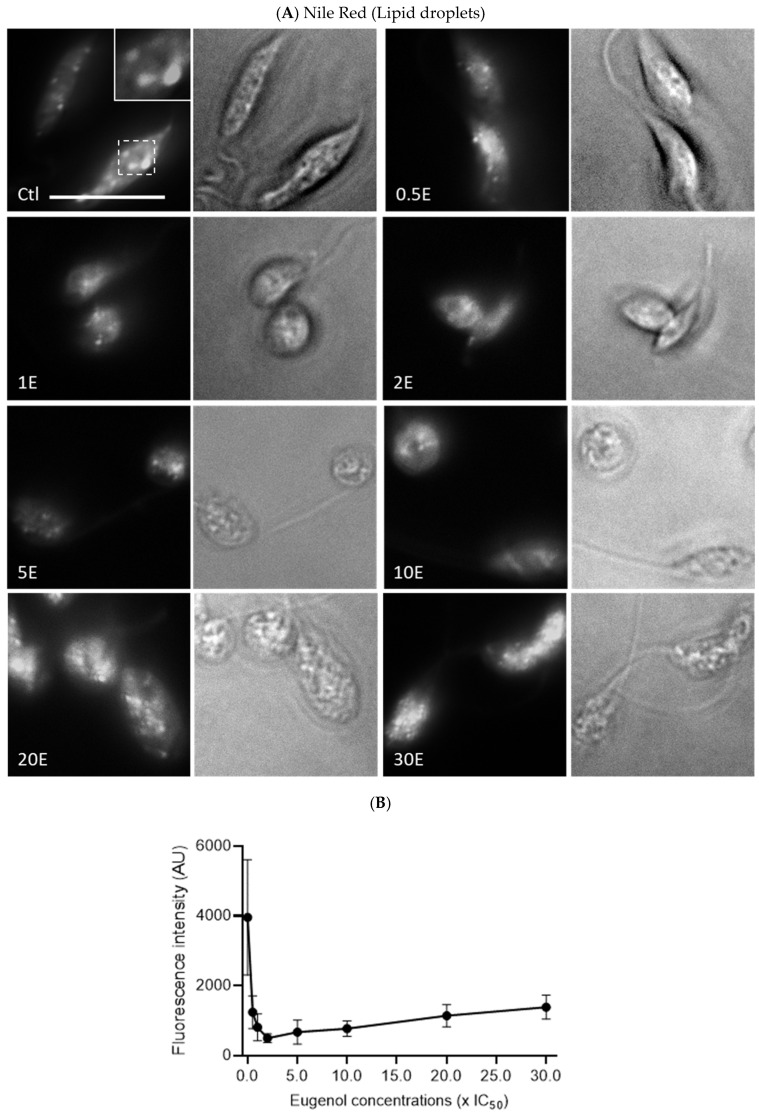
Eugenol decreases lipid droplet labeling, assessed by Nile Red dye. (**A**) Representative images of lipid droplets by Nile Red fluorescence microscopy of *Lmm* treated for 24 h with 0.01% DMSO as control (Ctl) and eugenol at 0.5–30× IC_50_ (0.5–30E) on the left panel and corresponding brightfield image in right panel. Scale bars, 10 µm. (**B**) Image quantification. Values are presented ±SD and represent the average of two independent experiments with 4 to 6 images per condition.

In contrast, lipid droplets decreased significantly and dose dependently between 0.5× IC_50_ and 2–5× IC_50_, nearly eightfold compared to the untreated promastigotes, before re-increasing (Figure 8A,B).

## 3. Discussion

The effect of eugenol on *Lmm* promastigotes was studied to gain a better understanding of its mode of action. Eugenol-induced rounding of promastigotes after 24 and 72 h of treatment at concentrations around IC_50_. An increase in membrane permeability and a decrease in fluidity in LUVs were observed at concentrations above 20× IC_50_, while an increase in the fluidity of *Lmm* membranes was observed at the same concentrations. The results showed that eugenol mainly induced necrosis rather than apoptosis (whether early or late) as a cell-death mechanism, at the various concentrations and incubation periods tested. Further investigation revealed that according to the Alamar Blue assay, metabolic activity related to NADH/NAD^+^ and cytochrome C oxidase was drastically reduced starting at 5× IC_50_. Lipid droplets were also reduced between 0.5–5× IC_50_ of eugenol while the abundance of acidocalcisomes was not affected. Throughout our experiments, we chose to test eugenol at increasing concentrations up to the ones allowing for a significant effect on the activities studied, while amphotericin B was tested at lower concentrations for which it was already known to have a significant effect. This allowed for the measurement of the difference in the extent and efficacy of eugenol compared to known reference drugs. 

The change in promastigotes morphology from elongated to round and swollen was previously observed in the shape of *L. donovani* promastigotes after treatment with a eugenol-rich *Syzygium aromaticum* oil at 100 µg/mL and eugenol emulsions [17,37] as well as several other antileishmanial compounds.

Feret ratios obtained after eugenol treatment of *Lmm* indicate a reduction of body length, describing the shift from elongated to rounded promastigotes. Although the morphology was affected by concentrations near the IC_50_ of eugenol, membrane disruption was neither the cause nor the end result at these concentrations. Eugenol only showed a 10% increase in LUVs permeability at concentrations above 30× IC_50_. These observations concur with those of a previous publication showing that, at a MIC concentration (or 100× *Lmm* IC_50_), eugenol was found to induce 12.5% leakage in *Saccharomyces cerevisiae* membrane-like LUVs [38]. LUVs composition differs between *S. cerevisiae* and *Lmm* models, though, in both cases, weak LUV membrane permeability was observed at high concentrations (>20× IC_50_). Nonetheless, amphotericin B is known to target *Lmm* membranes and showed a comparable increase in the permeability to eugenol, although at concentrations approximately 30 times lower than eugenol (0.25× IC_50_ instead of 7.5× IC_50_). This indicates that, although the permeabilizing effect is present with high doses of eugenol, the extent of the disruption might be comparable to that of amphotericin B. Furthermore, a closer investigation of the impact of eugenol on the membranes highlighted a different mode of action than that of amphotericin B. Indeed, the activity of eugenol does not appear dependent on the presence of sterols (ergosterol or cholesterol) in LUVs, while amphotericin B is known (and shown) to preferentially target ergosterol-containing LUVs [19].

Similar to permeability, membrane fluidity is only significantly affected by eugenol at concentrations above 20× IC_50_. In this regard, loss of membrane integrity mainly occurs at high concentrations of the compound and leads to necrosis. The high concentrations needed to observe membrane disruption suggest the role of other membrane components than the lipids included in the LUVs. Furthermore, the opposite effects on the membrane fluidity of LUVs versus *Lmm* membranes were observed. This could be due to differences in the components present in artificial membranes and membranes from parasites, and especially the presence of membrane proteins. Furthermore, differences in experimental conditions such as the duration of incubation (30 min for LUVs experiments and 24 h for *Lmm* plasma membranes) could also explain the differences in results since the compounds could be differently inserted in the membrane because of a lesser or greater duration of contact with the membranes, thus modifying the DPH location [39]. 

Intracellularly, nuclear DNA fragmentation might have occurred while membranes remained intact, as previously observed for *L. donovani* promastigotes treated with 4 mM H_2_O_2_ for 6 h [40]. We here showed that acidocalcisomes revealed by the pyrophosphatase immunolabeling were not significantly affected by eugenol concentrations. Nonetheless, eugenol may still target Ca^2+^. Indeed, an eugenol-rich *S. aromaticum* oil caused depolarization of the mitochondrial membrane [37], while an eugenol-rich essential oil from *Ocimum gratissimum* induced mitochondrial swelling after 72 h of treatment [41]. SQ109 is a drug inhibiting *Leishmania donovani* proliferation and was shown to cause deregulation of Ca^2+^ in mitochondria, via disruption of the mitochondrial membrane potential and acidocalcisomes, by their alkalinization [42]. These findings agree with the Alamar Blue assay results as cytochrome c oxidase is involved in mitochondrial energetic mechanisms.

This study also shows for the first time that eugenol dose-dependently decreases the concentration of intracellular lipid inclusions between 0.5 and 5× IC_50_. Miltefosine was shown to cause an overall depletion of lipid metabolites after 7.5 h of treatment, which was suggested to be caused by leakage and, thus, impairment of cellular membranes [43]. However, we observe a sharp decline in lipid droplets at concentrations for which membrane permeability and fluidity are not or very mildly affected. Lipids have a variety of functions in *Leishmania* sp. and some are linked to the structural needs of the parasites while others are linked to the metabolic needs as energy storage for the cells [36]. This could be due to the conversion and subsequent use of lipids to fulfill energetic needs in stress conditions induced by exposure to eugenol.

## 4. Materials and Methods

### 4.1. Chemicals and Reagents

Eugenol, amphotericin B, calcein, sephadex G-50, LH-20, ergosterol (Erg-), pentamidine isethionate, propidium iodide (PI), and 1,6-diphenyl-1,3,5-hexatriene (DPH) were obtained from Sigma-Aldrich (Bornem, Belgium). Miltefosine was obtained from Santa Cruz Biotechnology (Heidelberg, Germany). Calcein-AM included in a live/dead Viability/Cytotoxicity Kit for mammalian cells, Alamar blue and goat anti-Guinea Pig IgG Secondary Antibody, Alexa Fluor™ 488 were obtained from Thermo Fisher Scientific (Merelbeke, Belgium). The 1-palmitoyl-2-oleoyl-*sn*-glycero-3-phosphocholine (POPC), 1-palmitoyl-2-oleoyl-*sn*-glycero-3-phosphoethanolamine (POPE), l-α-phosphatidylinositol (PI—Liver, Bovine), and cholesterol (Chol—Ovine Wool) were purchased from Avanti Polar Lipids Inc. (Alabaster, AL, USA). All organic solvents used were from VWR (Leuven, Belgium).

### 4.2. Scanning Electron Microscopy

*L. mexicana mexicana* promastigotes at the density of 10^5^ cells/mL were treated for 72 h with 0.5, 1, or 2× IC_50_ of eugenol (IC_50_ = 16.59 µM), or IC_50_ of amphotericin B (IC_50_ = 0.25 µM). After centrifugation at 600× *g* for 10 min, parasites were washed 2 times with phosphate-buffered saline (PBS, pH 7.4) and once with cacodylate buffer 0.1 M (pH 7.4). They were then immobilized on poly-L-lysine-coated coverslips for 5 min at room temperature. After washing 3 times with cacodylate 0.1 M (pH 7.4) to remove the excess of free-floating *Leishmania*, the coverslips were incubated in 1% glutaraldehyde in cacodylate 0.1 M (pH 7.4) to crosslink the fixed parasites on the poly-L-lysine coating. The parasites were postfixed with 1% osmium tetroxide in a cacodylate buffer for 2 h at 4 °C and washed in water to eliminate traces of the remaining osmium tetroxide, then dehydrated in a graded series of ethanol, critical-point dried, and coated with 10 nm of gold. Samples were observed with a CM12 Philips electron microscope at 80 kV.

### 4.3. Preparation of Large Unilamellar Vesicles (LUVs) Mimicking Leishmania Plasma Membrane

Based on the lipid profile of the isolated surface membrane of *L. donovani* promastigotes reported by Wassef et al. [44], LUVs composed of PC:PE:PI:Erg (10:26:12:15) were prepared using the extrusion technique with the Avanti Mini Extruder (Avanti Polar Lipids, Inc., Alabaster, AB, USA) [45]. Briefly, lipids were dissolved in chloroform to obtain a stock solution of 25 mg/mL and then mixed in the required ratio. The lipid film, which was formed after evaporating the solvent under a vacuum with a Buchi Rotavapor R-200, rotary evaporator (Büchi Labortechnik AG, Flawil, Switzerland), was hydrated by Tris 10 mM and NaCl 159 mM at pH 7.4. The suspension was submitted to five cycles of freezing–thawing to obtain multilamellar vesicles (MLVs). The MLVs were extruded at 45 °C, 19 times through two polycarbonate Nucleopore Track Etch membrane filters (pore size = 100 nm) (Whatman Nucleopore, Corning Costar Corp., Badhoevedorp, The Netherlands) to obtain LUVs. Their size (average ± 150 nm) and the polydispersity index (PDI < 0.2) were controlled via a dynamic light-scattering technique using Zetasizer Nano ZS (Malvern Instruments, Malvern, UK). The phospholipid content was measured by phosphorus assay [46] and then adjusted to a final concentration of 5 μM.

To study the selective role of ergosterol, it was omitted in the preparation of some liposomes or replaced by cholesterol.

### 4.4. Calcein Release Assay

The leakage of calcein entrapped inside LUVs at the self-quenching concentration induced by a permeabilizing agent can be measured by fluorescence increase as calcein is diluted. For this permeability study, calcein, after purifying using column chromatography with sephadex LH-20, was diluted to the required concentration (73 mM) in the 10 mM Tris-HCl buffer solution at pH 7.4. This solution was then used for the hydration of the lipid film. The unencapsulated calcein was removed using the minicolumn centrifugation technique with sephadex G-50. Calcein-filled LUVs were incubated with eugenol or the reference drug amphotericin B, for 15 min at concentrations ranging from 0.5× IC_50_ to 40× IC_50_. Triton X 0.1% was used to induce 100% of the calcein leakage. After measuring the fluorescence intensity (excitation wavelength of 472 nm, emission wavelength of 512 nm) using a Perkin Elmer LS 30 Fluorescence Spectrophotometer (Perkin-Elmer Ltd., Beaconsfield, UK), the percentage of calcein released was calculated using the formula:

(1) $$\%\;calcein\;release=\frac{F_{t}-F_{ctrl}}{F_{tot}-F_{ctrl}}\times 100$$

F_t_: fluorescence signal measured in the presence of compound;
F_ctrl_: fluorescence signal measured for LUVs control;
F_tot_: fluorescence signal measured in the presence of Triton X-100;
Results are means of three independent experiments in triplicates.

### 4.5. DPH Fluorescence Anisotropy Assay

Fluorescence anisotropy values of DPH (r) can be used to probe the effect of a compound on membrane fluidity as the probe locates in the hydrophobic core of the lipid bilayer of the membrane and is sensitive to changes in the lipid acyl chain physical properties [47].

In LUVs- During the LUVs preparation process, DPH 2 mM in tetrahydrofuran was mixed with lipids at the molar ratio of 1:200 (DPH: lipid) before evaporating the solvents. The following steps were done as previously described in the section “Preparation of LUV mimicking *Leishmania* plasma membrane”. 

DPH-containing LUVs in a Tris HCl/NaCl buffer were then incubated with eugenol or amphotericin B for 30 min in the dark at the previously prepared concentrations. DPH was excited at 365 nm, and emission was read at 425 nm using an LS55 luminescence spectrometer connected to a circulating water bath to maintain the temperature at 28 °C [48].

DPH fluorescence anisotropy was determined using the equation:

(2) $$r=\frac{I_{VV}-G\times I_{VH}}{I_{VV}+2\times G\times I_{VH}}$$

I_VV_ and I_VH_: the fluorescence intensities with the excitation (wavelength of 365 nm) and emission (wavelength of 425 nm) polarization filters in vertical (v) and horizontal (h) orientations, respectively.

G: an inherent factor to the spectrophotometer used. 

In *Lmm*- Samples were prepared as described by Xu et al. [49]. Briefly, parasites at the density of 10^7^ cells/mL were treated for 24 h with eugenol (in the range of 1–40 × IC_50_) with IC_50_ 24 h = 5× IC_50_ 72 h, or reference drugs amphotericin B (in the range of 0.5–5× IC_50_) with IC_50_ 24 h = IC_50_ 72 h, and miltefosine (in the range of 1–10× IC_50_) with IC_50_ 24 h = 25.09 µM = 2× IC_50_ 72 h, then washed once with PBS, and resuspended in 1 mL of PBS. Then, 2 mM DPH in tetrahydrofuran were added to a final concentration of 0.5 μM and incubated in the dark for 20 min at 28 °C (the concentration of tetrahydrofuran in *Leishmania* suspension was 0.025%).

DPH parameters are the same as previously described and Equation (2) was used to determine DPH anisotropy.

Results of the DPH anisotropy for both LUVs and *Lmm* are means of three independent experiments in triplicates. 

### 4.6. Flow-Cytometry Analysis

*Lmm* promastigotes at the density of 10^7^ cells/mL were treated for 8 h, 16 h, and 24 h with eugenol in the range of 1–40× IC_50_ (IC_50_ 8, 16 and 24 h = 5× IC_50_ 72 h), or by two reference drugs, amphotericin B in the range of 0.5–5× IC_50_ (IC_50_ 8, 16 and 24 h = IC_50_ 72 h), and miltefosine between 1 and 10× IC_50_ (IC_50_ 24 h = 2× IC_50_ 72 h). Treated parasites were collected by centrifugation at 600× *g* for 10 min. After washing once with PBS, *Lmm* promastigotes were resuspended in 1 mL of PBS-containing calcein-AM 0.1 μM and PI 3 μM, then incubated for 15 min at room temperature in the dark. A total of 50,000 events per sample were acquired in a FACSCalibur cytometer using 488 nm excitation. Green fluorescence emission for calcein (530/30 bandpass) and red fluorescence emission for PI (610/20 bandpass) were measured.

### 4.7. Alamar Blue Assay

Using Alamar blue to analyze the effect of eugenol on the metabolic activity of *L. mexicana mexicana* promastigotes, parasites at the density of 10^7^ cells/mL were incubated with eugenol, amphotericin B, or miltefosine at the same range of concentrations as the flow cytometry experiment. After 20 h of incubation at 28 °C, Alamar blue (diluted 1:1 in PBS) was added and parasites were further incubated for 4 h at a final concentration of 4.5%. Fluorescence was measured on a spectrofluorimeter (SpectraMax-Molecular Devices, Berkshire, UK) (excitation wavelength of 530 nm, emission wavelength of 590 nm). The percentage of metabolic activity was calculated by comparing fluorescence values to those reported for the nontreated group.

### 4.8. Nile Red Labelling and Image Quantification

*Lmm* promastigotes at 10^7^ cells/mL were treated for 24 h with eugenol at concentrations between 0.5 and 30× IC_50_ (IC_50_ 24 h = 5× IC_50_ 72 h). The parasites were then centrifuged at 600× *g* for 10 min. The pellet was washed twice with PBS 1X, then fixed in 4% PFA. Then, 10 µL of the sample were mixed with 30 µL of a 10 µg/mL Nile Red solution in acetone and incubated for 10 min. The mixture was observed in a Zeiss LSM980 microscope. Quantification of *Lmm* morphology was obtained by measuring 4–6 images from two independent experiments for the Feret maximum diameter (accounting for length) and the Feret minimum diameter (accounting for width). The Feret ratio of *Lmm* was then calculated as follows:

$${Feret}_{ratio}=\frac{{Feret}_{min}}{{Feret}_{max}}$$

Similarly, quantification of the Nile Red fluorescence was done by measuring the mean green intensity in 4–6 images per concentration of eugenol after manually drawing the *Lmm* contour using confocal images in black and white. The results were obtained from 2 independent experiments. 

### 4.9. Anti-TbVP1 Labelling and Image Quantification

*Lmm* promastigotes at the density of 10^7^ cells/mL were treated for 24 h with eugenol at 1–30× IC_50_ (IC_50_ 24 h = 5× IC_50_ 72 h). Treated parasites were centrifuged at 600× *g* for 10 min. After washing twice with PBS, *Lmm* promastigotes were resuspended in 1 mL of PBS. Cells at 3 × 10^7^ Lmm/mL were then transferred to a 24-well poly-l-lysine-coated plate. After several washes in PBS 1X, they were fixed with 4% formaldehyde in 0.1 M phosphate buffer for 20 min at room temperature. Permeabilization was done in 0.1% Triton-X100 for 5 min at room temperature. After several washes in Q-PBS (1% bovine serum albumin, lysine at 1 mg/mL, 0.01% saponin, and 0.025% azide in PBS 1X), the cells were incubated with anti-TbVP1 primary antibodies [50] for 1 h and then with fluorescent Alexa Fluor 488 goat antiguinea pig secondary antibodies for 1 h. The coverslips were mounted in prolong gold and left to polymerize for 24 h. The images were taken on a Zeiss LSM980 microscope. Quantification was obtained by measuring the mean green intensity of *Lmm* in 4–6 images per concentration of eugenol after manually drawing the *Lmm* contour using confocal images in black and white. The results were obtained from 1 experiment.

### 4.10. Statistical Analyses

All statistical tests were performed on GraphPad Prism 9.4.1. One-way ANOVA with Dunnett’s post tests to compare the control versus compound-treated LUVs or *Leishmania* promastigotes, * *p* < 0.05; ** *p* < 0.01, *** *p* < 0.001, and **** *p* < 0.0001.

## 5. Conclusions

The results gave a better understanding of the processes involved in eugenol-mediated antileishmanial activity. Indeed, only the immunomodulatory effect of eugenol on mice with visceral leishmaniasis had been previously characterized. For the first time, it has here been demonstrated that eugenol, at concentrations close to its IC_50_, lowers the metabolic activity of *Leishmania* promastigotes via a dose-dependent decrease of lipid droplets without impacting membrane integrity. Eugenol also induces morphological alterations, namely rounding and swelling of the promastigotes at such concentrations. Furthermore, eugenol has been demonstrated to not target membrane sterols, such as ergosterol, unlike amphotericin B. Further studies on the molecular targets of eugenol could prove interesting to propose its use in combination therapy with currently existing drugs.

## Data Availability

The data presented in this study are available in the manuscript.

## Sample Availability

Samples of the compounds are not available from the authors.
